# Enhancing cognitive accessibility in existential ­psychotherapy for adults with brain tumors: A patient-informed approach to intervention adaptation

**DOI:** 10.1093/noajnl/vdag087

**Published:** 2026-04-02

**Authors:** Ashlee R Loughan, Autumn Lanoye, Alex Davies, Amber Fox, Giuliana Zarrella

**Affiliations:** School of Medicine, Virginia Commonwealth University, Richmond, Virginia (A.R.L., A.L.); School of Medicine, Virginia Commonwealth University, Richmond, Virginia (A.R.L., A.L.); Central Virginia Virginia Healthcare System, Richmond, Virginia (A.D.); Department of Psychology, Virginia Commonwealth University, Richmond, Virginia (A.F., G.Z.); Department of Psychology, Virginia Commonwealth University, Richmond, Virginia (A.F., G.Z.)

**Keywords:** brain tumor, psychotherapy, cognitive adaptations

## Abstract

Individuals with primary or secondary brain tumors experience *double vulnerability*—significant emotional distress alongside cognitive decline. These cognitive changes often prohibit their inclusion in psychotherapeutic interventions due to restrictive eligibility criteria; however, simple accommodations including appointment reminders, therapy topic refreshers, prior session summaries, and additional time for processing information may support cognitive accessibility and enable engagement in interventions that promote well-being. Implementing such accommodations is vital to promoting neuro-oncology inclusion in psychotherapeutic interventions and meeting the needs of this underserved population.

Many adults with brain tumors experience *double vulnerability*—significant emotional distress alongside cognitive decline. These cognitive changes often prohibit their inclusion in psychotherapeutic intervention research due to restrictive eligibility criteria; however, simple accommodations may support cognitive accessibility and enable engagement in interventions that promote well-being. This analysis aimed to identify and evaluate evidence-informed accommodations, using a patient-informed approach, to support the neurocognitive needs of patients with brain tumors to enhance their engagement in psychotherapeutic interventions.

We conducted a series of feasibility studies, following the NIH ORBIT Model,^1^ of Managing Cancer and Living Meaningfully (CALM) in patients with primary or secondary brain tumors. CALM is a brief (6-session) semi-structured supportive-expressive psychotherapy grounded in relational, attachment, and existential theory, developed to help patients with advanced cancer adapt to the challenges of their disease and its treatment, manage distress while remaining engaged in life, and increase preparedness for end-of-life.^2^ While empirical research has shown CALM to be effective in treating and preventing psychological distress across various advanced cancer populations,^3^ all trials to date investigating CALM have purposely excluded individuals with brain tumors due to presumed cognitive impairment—a common assumption that frequently goes unopposed and causes direct discrimination against those who may need the intervention the most. We are the first to empirically evaluate CALM in individuals with brain tumors, with the goal of identifying which evidence-informed cognitive accommodations may improve participants’ ability to fully engage in this psychotherapeutic intervention.

First, in 2 single-arm pilot trials,^4^^,^^5^ 18 participants with either primary (*n *= 9) or secondary brain tumors (*n *= 9) completed exit interviews following CALM and rated which accommodations (Table 1)^6^^,^^7^ would have supported their cognitive limitations and enhanced intervention engagement. Accommodations endorsed by ≥60% of participants were selected for incorporation into the next phase of the research program. These recommended accommodations were then implemented as standardized intervention procedures in a subsequent randomized pilot trial evaluating the feasibility of CALM in patients with primary or secondary brain tumors (*N* = 58). The accommodations themselves were not randomized across participants; rather, all participants receiving CALM experienced the same set of incorporated cognitive supports. Following completion of the intervention, participants completed a brief evaluation indicating whether each accommodation was beneficial in supporting their engagement in therapy (*N* = 29; dichotomous response: yes/no). Eligibility criteria across trials were (1) age ≥18 y, (2) English-speaking, (3) self-reported diagnosis of primary or secondary brain tumor, (4) 2 weeks post-surgical cranial resection or biopsy, (5) indication of psychological distress (depressive symptoms on Patient Health Questionnaire—9 item ≥10 or death anxiety severity on Death and Dying Distress Scale ≥15), (6) absence of major neurocognitive impairment (≥20 on the Telephone Interview for Cognitive Status [TICS]), and (7) reliable internet access to participate in the telehealth intervention.

**Table 1.** Cognitive accommodations recommended and found beneficial.

Cognitive accommodation	Single-arm recommended (*N* = 18)	Randomized pilot found benefit (*N* = 29)
Appointment reminders	100%	97%
Therapy topic refreshers	94%	83%
Prior session summaries	89%	97%
Visual handouts	78%	66%
Time for content consolidation	78%	86%
Notepads	67%	45%
Survey support	27%	—

Participants from the single-arm pilot trials (*N *= 18) were 89% female, 83% White, and mostly middle-aged (*M *= 48.8 y; SD = 15.8; range: 19-80). The majority (89%) had intact cognitive functioning based on a TICS score >31; 11% had TICS scores falling into the mild cognitive impairment range. Ratings of recommended accommodations are provided in Table 1. Given the minimal recommendation for survey ­support (<60%), this accommodation was not carried forward. Participants from the subsequent randomized pilot trial (*N *=* 58*) were 67% female, 88% White, and mostly middle-aged (*M *= 51.8 y; SD = 11.3; range: 26-71). The majority (86%) of participants had intact cognitive functioning based on a TICS score >31; 14% of participants had TICS scores falling into the mild neurocognitive impairment range. Ratings of incorporated accommodations shown to be proving beneficial are provided in Table 1.

The most recommended evidence-informed cognitive accommodations by participants with primary or secondary brain tumors were (1) more frequent appointment reminders in participants’ preferred form (eg email, phone call, text) to support session attendance, (2) therapy topic refreshers and prior session summaries to support memory, and (3) additional time to assist with slower processing and support comprehension during conversation. Patients received 2 automated appointment reminders via email, one at the end of the prior session and another 24 h beforehand. During each session, interventionists reviewed the core concepts (eg “*mentalization*”—capacity to reflect on one’s own and others’ mental states—and “*dual awareness*”—the ability to hold 2 seemingly opposing realities—life’s possibilities and the eventuality of dying—simultaneously) and domains of CALM (ie symptom management and communication with health care providers, changes in self and relations with others, spirituality and sense of meaning, and the future, hope, and mortality) and provided an overview of the major themes discussed during the prior session. Finally, all interventionists received specialized training in the neurocognitive deficits associated with primary or secondary brain tumors and their treatment, as well as corresponding cognitive strategies to appropriately support the unique and diverse needs of this population. For instance, interventionists structured sessions to allow additional time for patients with speech latency or word-finding difficulty to respond and for repetition or cueing/prompting by the interventionist to support encoding/retrieval for patients with memory decline. Survey support was only minimally recommended and never requested by the study team. This aligns with the high feasibility shown in patient-reported outcome measure completion across neuro-oncology trials.^8^^,^^9^ Also, while notepads were highly recommended in the single-arm pilots, they were considered only moderately beneficial in the randomized trial. Targeted adaptations, such as providing an interactive participant workbook rather than a blank notepad, which offers additional structure, encourages dialogue, and promotes engagement with therapeutic techniques, may bolster the benefit for participants.^10^

Implementing supportive cognitive strategies is vital to promoting greater inclusion of neuro-oncology populations in psychotherapeutic interventions and addressing the needs of this underserved group. The present findings suggest that individuals with brain tumors can engage in psychotherapeutic interventions when simple, evidence-informed cognitive accommodations are integrated to support common neurocognitive challenges. These results highlight a way to reduce barriers that have historically limited participation of patients with brain tumors in psycho-oncology research. With appropriate cognitive accommodations integrated, individuals with brain tumors may be able to learn and apply strategies to reduce psychological distress and support well-being.

## Data Availability

Data will be made available upon reasonable request to corresponding author.

## References

[1] Czajkowski SM , Powell LH, Adler N, et al From ideas to efficacy: the ORBIT model for developing behavioral treatments for chronic diseases. Health Psychol. 2015;34:971-982. 10.1037/hea0000161 25642841 PMC4522392

[2] Rodin G , Hales S. Managing Cancer and Living Meaningfully: An Evidence-Based Intervention for Cancer Patients and Their Caregivers. Oxford University Press, New York, NY; 2021.

[3] Rodin G , Lo C, Rydall A, et al Managing cancer and living meaningfully (CALM): a randomized controlled trial of a psychological intervention for patients with advanced cancer. J Clin Oncol. 2018;36:2422-2432. 10.1200/JCO.2017.77.1097 29958037 PMC6085180

[4] Loughan AR , Lanoye A, Willis K, et al Managing cancer and living meaningfully in adults with brain metastases: a NIH ORBIT model phase II feasibility and proof-of-concept trial. Neurooncol Pract. 2025;12:271-280. 10.1093/nop/npae097 40110068 PMC11913651

[5] Loughan AR , Willis KD, Braun SE, et al Managing cancer and living meaningfully (CALM) in adults with malignant glioma: a proof-of-concept phase IIa trial. J Neurooncol. 2022;157:447-456. 10.1007/s11060-022-03988-8 35437687 PMC9909556

[6] Dawson P , Guare R. Smart but Scattered: Second Edition: The Revolutionary Executive Skills Approach to Helping Kids Reach Their Potential. The Guilford Press, New York, NY; 2009.

[7] Meltzer L. Executive Function in Education: Second Edition: From Theory to Practice. The Guilford Press, New York, NY; 2007.

[8] Dirven L , Vos ME, Walbert T, et al Systematic review on the use of patient-reported outcome measures in brain tumor studies: part of the response assessment in Neuro-Oncology Patient-Reported outcome (RANO-PRO) initiative. Neurooncol Pract. 2021;8:417-425. 10.1093/nop/npab013 34277020 PMC8278354

[9] Renovanz M , Hechtner M, Kohlmann K, et al Compliance with patient-reported outcome assessment in glioma patients: predictors for drop out. Neurooncol Pract. 2018;5:129-138. 10.1093/nop/npx026 31385978 PMC6655376

[10] Schumacher RB , Wantz RA, Taricone PF. Constructing and using interactive workbooks to promote therapeutic goals. Elem Sch Guid Couns. 1995;29:303-309.

